# Globalization and glocalization in graduate medical education: the ACGME international experience in Qatar

**DOI:** 10.3389/fmed.2026.1919360

**Published:** 2026-08-31

**Authors:** Shireen Suliman, Abdulla Al Naimi

**Affiliations:** 1 Department of Medical Education, Hamad Medical Corporation, Doha, Qatar; 2 College of Medicine, QU Health, Qatar University, Doha, Qatar; 3 Weill Cornell Medicine - Qatar, Doha, Qatar; 4 School of Health Professions Education, Maastricht University, Maastricht, Netherlands; 5 Department of Urology, Hamad Medical Corporation, Doha, Qatar

**Keywords:** Accreditation Council for Graduate Medical Education, Accreditation Council for Graduate Medical Education–International, globalization, glocalization of learning, graduate medical education, Qatar

## Abstract

A fundamental challenge associated with the implementation of global benchmarks arises from reconciling standardized international expectations with diverse local contexts, as differences in healthcare systems, cultural norms, and resource availability often limit the direct applicability of externally developed standards and require context-sensitive adaptation. As the globalization of graduate medical education continues to advance, initiatives such as the international adoption of milestone-based frameworks demonstrate that global convergence is feasible when accompanied by deliberate localization. In Qatar, following the establishment of GME in the form of residency and fellowship programs, there was a clear need to introduce an international accreditation body to support the development, standardization, and formalization of GME within the national context. With the adoption of Accreditation Council of Graduate Medical Education International (ACGME-I) accreditation, it is important to pay attention to the processes by which accreditation standards are contextualized and aligned with local educational, clinical, and health-system realities. This piece of work aims to explore how ACGME-International accreditation standards are contextualized within local GME systems, using the Qatar experience as a case example to examine institutional motivations, adaptation strategies, and perceived alignment with local educational and clinical realities. We aim to underscore how effective accreditation depends not only on compliance with global standards but also on their thoughtful integration within the realities of the local educational and health-care context.

## Introduction

How can institutions uphold international standards whilst remaining responsive to distinct local contexts? A fundamental challenge associated with the implementation of global benchmarks arises from reconciling standardized international expectations with diverse local contexts, as differences in healthcare systems, cultural norms, and resource availability often limit the direct applicability of externally developed standards and require context-sensitive adaptation. Nevertheless, international accreditation, such as that offered by the Accreditation Council for Graduate Medical Education–International (ACGME-I), is widely regarded as valuable and has been increasingly adopted globally. The objectives underpinning ACGME-I accreditation are multifaceted, encompassing standardization, quality assurance, and capacity building (1). The adoption of ACGME-I accreditation has been associated with several benefits, including improved consistency across training programs, enhanced educational quality, and strengthened governance structures (2). ACGME-I provides a unified framework for residency and fellowship programs, facilitating the transition toward outcomes-based medical education and clarifying the competencies expected of graduating trainees (3). Consequently, graduates are equipped to demonstrate proficiency across the six core competency domains, including Patient Care, Medical Knowledge, Practice-Based Learning and Improvement, Professionalism, Interpersonal and Communication Skills, and Systems-Based Practice (4, 5). In addition, ACGME-I accreditation has been associated with improved trainee-perceived learning environments enhanced workforce mobility, and increased opportunities for international collaboration and benchmarking. Accredited institutions further benefit from access to structured evaluation systems, faculty development initiatives, and mechanisms that support continuous quality improvement (6, 7). While the Accreditation Council for Graduate Medical Education (ACGME) is structured around the U. S. health-care and regulatory environment, ACGME-I was developed to accredit programs outside the United States and therefore incorporates flexibility to accommodate diverse local health systems, cultural contexts, and disease patterns while maintaining core educational standards (2). This flexibility, supported by internationally representative peer-review processes, positions ACGME-I as a global framework intended for local interpretation and adaptation rather than uniform application, consistent with principles of glocalization (8).

While the ACGME-I was intentionally designed to provide flexibility and accommodate local health systems, cultural contexts, and disease patterns, the mere presence of adaptable standards does not, in itself, guarantee meaningful alignment. While international accreditation delivers clear and well-documented benefits, its effectiveness ultimately depends on how global standards are interpreted, adapted, and enacted within local contexts. Healthcare delivery is deeply embedded in cultural traditions, patient expectations, and broader societal values, and effective training programs must reflect these realities. Institutions also vary significantly in terms of infrastructure, technological capacity, and workforce composition, necessitating a collaborative approach between accreditation bodies and local institutions to co-develop and adapt standards without compromising their core integrity (9). Moreover, national health priorities, legal frameworks, and workforce needs differ across settings; alignment with local health-system objectives is therefore essential to ensure relevance and sustainability. This calls for the principle of *glocalization*—the intentional integration of global frameworks with local realities—to promote relevance, legitimacy, and effectiveness (10, 11). As the globalization of graduate medical education (GME) continues to advance, initiatives such as the international adoption of milestone-based frameworks demonstrate that global convergence is feasible when accompanied by deliberate localization (12). Originating in the late 1980s within business discourse and later adopted in higher and medical education, glocalization emphasizes integration rather than imposition (13). Glocalization describes the dynamic integration of global and local influences while preserving the unique contributions of diverse cultural and institutional contexts (14). In education, it informs theory and practice by fostering approaches that are contextually relevant, inclusive, equitable, and responsive to cultural diversity (15). In practice, this involves adopting global competencies to local context incorporating them within curricula while tailoring teaching approaches, clinical exposures, assessment practices and community engagement to address local health challenges. It also requires ethical sensitivity, rejecting one-size-fits-all models that risk cultural erasure or inequity. Ultimately, accreditation frameworks should empower local educators to interpret and apply standards through their contextual expertise, supported by collaborative governance structures that balance accountability with flexibility.

Against this conceptual backdrop of glocalization—the deliberate integration of global standards with local realities—the Qatari experience provides a salient case for empirical examination. In Qatar, following the establishment of GME in the form of residency and fellowship programs, there was a clear need to introduce an international accreditation body to support the development, standardization, and formalization of GME within the national context. The adoption of ACGME-I accreditation not only addressed these structural needs but also conferred regional and international recognition upon Hamad Medical Corporation (HMC), positioning it as the first institution in the region—and the second globally—to achieve such accreditation (16). Despite these achievements, existing literature has predominantly emphasized the benefits and outcomes of international accreditation (7, 17), with limited attention given to the processes by which accreditation standards are contextualized and aligned with local educational, clinical, and health-system realities. This gap is particularly important given the global rise of ACGME-I and the increasing institutional desire for international accreditation, which now includes 37 accredited institutions worldwide. Understanding how global standards are interpreted, adapted, and operationalized within specific contexts is therefore critical to assessing their impact and facilitating smoother, more sustainable adoption. Accordingly, this piece of work aims to explore how ACGME-International accreditation standards are contextualized within local GME systems, using the Qatar experience as a case example to examine institutional motivations, adaptation strategies, and perceived alignment with local educational and clinical realities.

## Integration of ACGME-I institutional and foundational requirements with glocalization in HMC

Table 1 illustrates how ACGME-I institutional and program requirements (1, 18) were integrated within GME training programs in Qatar through a glocalization approach. This integration unfolded progressively and was largely driven by contextual needs, occurring both proactively and reactively rather than being fully established prior to implementation. The approach reflects an adaptive process in which international standards were interpreted and aligned with local educational, clinical, and health-system realities. Specifically, the table maps ACGME-I institutional and program requirements against the strategies used to contextualize these standards within the Qatari GME landscape.

**Table 1:** ACGME-I institutional and program requirements were integrated within GME training programs in Qatar through a glocalization approach.

ACGME-I Institutional and program requirements	Requirements integration within GME programs
Institutional requirements – organization and responsibilities	
National commitment and regulatory support	National commitment to ACGME-I accreditation is demonstrated through formal endorsement by Qatar’s Minister of Public Health and HMC’s Managing Director.
Institutional governance	Centralized governance of GME at HMC was implemented to enable coordinated oversight across a large network of hospitals and facilities.
Institutional governance	The DIO role at HMC was structured to align with ACGME-I requirements while integrating with senior institutional leadership to ensure effective oversight and decision-making access.
Training sites	PLA implementation at HMC was adapted within a unified institutional structure to maintain clarity of responsibility and educational oversight.
Clinical learning environment	Patient care accreditation at HMC was achieved through JCI recognition and adapted to align with the local Qatari context and national healthcare priorities.
Policy governance	Policy governance at HMC was operationalized through the integration of institutional policies with ACGME-I standards to ensure comprehensive oversight of residents and fellows.
Admissions policies	Admission policies prioritize Qatari nationals and require Arabic proficiency, while maintaining international competency standards aligned with local clinical practice.
Trainee selection process	Selection processes at HMC were standardized through central tools and the involvement of the GME office to ensure consistency, fairness, and transparency across programs. Candidate intake aligns with ACGME-I-approved complement numbers, with adjustments supporting national workforce development and resource availability.
Institutional requirement – the learning and working environment	
Trainee governance	Trainee engagement at HMC was enhanced through the establishment of a structured trainee council aligned with ACGME-I standards and local cultural context.
Reporting and confidentiality	Confidential communication and wellbeing initiatives at HMC were implemented to support safe feedback and trainee wellness while reflecting local cultural considerations.
Mental health support and counseling services	Wellbeing and mental health support at HMC were strengthened through culturally sensitive, accessible initiatives developed in collaboration with HR and staff engagement teams. Confidential counseling services and rapid response systems ensure timely support, aligning with ACGME-I standards while addressing local stigma and privacy concerns.
Institutional requirements – Graduate Medical Education Committee	
GMEC governance	GMEC governance at HMC was established to integrate institutional commitment to GME within structured oversight aligned with ACGME-I standards.
Program personnel and resources	
Program leadership	Program leadership and faculty eligibility at HMC were structured to align with ACGME-I requirements while allowing contextual recognition of local and regional certifications without compromising standards.
Faculty development	Faculty development at HMC was designed to integrate international collaboration with local capacity building to strengthen faculty competence and peer education. These initiatives cover areas such as teaching, assessment, mentorship, and addressing biases, bullying, and harassment in medical education, all tailored to the local context utilizing locally relevant case studies while maintaining universal principles.
Educational program	
Program structure and duration	Program structures remained aligned with ACGME-I standards while adapting to national certification and workforce requirements. Training duration was extended by one year in selected residency and fellowship programs to meet local and regional board certification and promotion requirements.
Curriculum design and rotations	Curricula aligned with the six ACGME core competencies while incorporating local disease patterns, patient demographics, cultural norms, and healthcare priorities. Additional research and specialty-specific rotations were introduced in selected programs to address departmental and workforce needs.
Educational activities	Teaching strategies incorporated culturally relevant case scenarios, simulation exercises reflecting common patient encounters in Qatar, multilingual communication workshops, and faculty development on professionalism, bias, bullying, and harassment using locally contextualized examples.
Procedural training	Procedural and case-log requirements at HMC were modified, following ACGME-I approval, to reflect local patient populations and service patterns. For example, selected gynecological and urological procedural thresholds were adjusted while maintaining competency-based standards.
Competency-based assessment	Competency-based evaluation at HMC is implemented through CCCs using a glocalized approach that integrates ACGME-I standards with local clinical and educational contexts. Structured tools, calibration processes, and faculty development ensure reliable, context-relevant assessments aligned with Milestones while reflecting local practice.
Specialty-specific evaluation tools	Assessment tools were adapted for specialties with unique learning environments (e.g., Hematology and Pathology) by developing supplementary evaluation methods appropriate for laboratory-based and consultative practice.
Program evaluation and continuous improvement	Program evaluation at HMC is designed to support continuous improvement while integrating ACGME-I requirements with local workforce needs, service demands, and institutional priorities. Evaluation outcomes are reviewed within governance structures such as the GMEC, ensuring accountability while enabling flexible, needs-based program enhancements

### Organization and responsibilities

ACGME-I requires the sponsoring institution to assume ultimate responsibility for all accredited GME programs. Following ACGME-I accreditation in 2012, HMC was designated as the sponsoring institution, effectively embedding accreditation into its strategic mandate as Qatar’s national public health provider. A formal commitment statement, signed by the Managing Director, the Chief Medical Officer and the Director of Medical Education of HMC underscores the nation’s dedication to aligning with the ACGME-I accreditation standards. HMC is an extensive institution that encompasses more than 15 hospitals and facilities throughout the state of Qatar. Governance of all GME programs has been centralized within HMC’s Medical Education Department, rather than being distributed among individual programs. This centralization allows for the development of institutional support mechanisms that are both locally relevant and aligned with international standards. Specifically, assessment practices, faculty development initiatives, and data management processes have been adapted to meet local needs while ensuring compliance with global best practices. This centralized model leveraged national and regional faculty development initiatives tailored to Qatar’s educational context and scale, enabling HMC to fulfil ACGME-I requirements while optimizing resource efficiency. The outcome was a locally sustainable system that upheld international standards without replicating resource-intensive models designed for different healthcare environments.

ACGME-I mandates an organized administrative system led by a Designated Institutional Official (DIO) with authority over all accredited programs. In HMC, this role was deliberately arranged to align with prevailing institutional leadership models. The DIO position was combined with senior executive and clinical leadership roles (such as the Director of Medical Education and the Deputy Chief Medical Officer for Education), ensuring direct access to decision-makers while maintaining the integrity of educational oversight. This structural adaptation while preserve the functional intent of the DIO role—authority, accountability, and oversight—it integrates it within a leadership model that is culturally and organizationally coherent within the local health system.

The ACGME-I requires that the sponsoring institution establishes Program Letters of Agreement (PLAs). At HMC, this requirement has been adapted contextually. Rather than duplicating agreements across its 15 hospitals, HMC has utilized its unified governance structure to mandate PLAs only for external institutions. This approach preserves the core purpose of PLAs—ensuring clarity of responsibility and educational oversight—while adapting the process to local operational realities. Additionally, all training sites are required to have accreditation for patient care. HMC achieved official Joint Commission International (JCI) accreditation for its hospitals in 2006. The organization adapted the JCI guidelines to align with the local Qatari context, incorporating culturally appropriate patient care practices, addressing the specific health needs of the population, and aligning healthcare services with national healthcare priorities. Since that time, HMC has consistently maintained and expanded its accreditations across its diverse network of hospitals, specialized care centers, and community services. This ongoing commitment to quality and culturally competent care underscores HMC’s dedication to enhancing healthcare delivery in Qatar.

#### Responsibilities for residents and fellows

ACGME-I requires the Sponsoring Institution to maintain comprehensive written policies governing residents and fellows. In HMC, this requirement was operationalized through the deliberate integration of HMC institutional policies with ACGME-I standards, reflecting an approach consistent with glocalization rather than replication of U. S. academic structures. Accordingly, specific GME policies are developed, approved, and periodically reviewed by Regulatory, Accreditation, and compliance Services team at HMC, with explicit alignment to HMC human resources bylaws and national workforce priorities. For example, policies addressing harassment and bullying were harmonized with existing HR protocols to ensure both compliance with ACGME-I expectations and consistency with local legal and organizational frameworks. Similarly, while duty hours and leave entitlements adhere to ACGME-I standards, local HR regulations allow unused leave to be compensated at the end of training rather than forfeited. This contextual adaptation preserves trainee well-being and program accountability while responding to local employment norms. Program-specific supervision policies further reflect glocalization, as they are aligned with overarching GME standards but tailored to accommodate variations in clinical rotations, service demands, and program-specific workloads.

The eligibility and selection of residents and fellows at both the institutional and program levels was also glocalized. While ACGME-I and program requirements emphasize transparent eligibility criteria, adequate resources, and fair recruitment processes, these standards were adapted in HMC to align with national workforce development goals. Admission criteria prioritize Qatari nationals in line with Qatarization, while upholding international competency standards. This approach integrates global benchmarks with local expectations, emphasizing service to Qatar’s health system and cultural awareness. Preference is given to graduates of Qatari medical schools, with Qatari nationals receiving the highest priority. In addition, Arabic language proficiency is mandated for most training programs, reflecting the linguistic realities of the patient population and clinical environment. Despite these local priorities, the selection process remains internationally competitive and aligned with ACGME-I competency expectations. The use of standardized selection tools, including a centrally developed Residency Interview Guide, along with the active involvement of GME office representatives during interviews, strengthens governance within GME processes. This strategy promotes consistency, fairness, and transparency across programs, while also enabling the systematic exchange and sharing of best practices at the institutional level. The number of selected candidates aligns with the approved complement numbers in accordance with ACGME-I procedures and standards. Any changes in the complement numbers—whether increases or decreases within the training programs—were primarily made to address the need for nationalization and to strengthen the national workforce while considering the available resources of each program.

#### The learning and working environment

The ACGME-I emphasizes the creation of a supportive learning and working environment for residents and fellows, essential for fostering their professional development and wellbeing. Tailored to the local context, HMC has integrated these standards in several initiatives. For instance, creating a trainee council forum allows residents and fellows to voice their concerns and provide feedback in a structured and safe manner. This forum not only aligns with ACGME-I guidelines but also resonates with Qatari cultural values that promote collective engagement and community support. Such forums can facilitate open dialog, allowing trainees to address issues while respecting the local hierarchical dynamics. Moreover, the development of confidential communication channels, such as a dedicated email system for raising concerns with the DIO without fear of retaliation, is another embodiment of glocalization. By anonymizing the feedback process, the program acknowledges local sensitivities around privacy and power dynamics, ensuring that trainees feel secure in voicing their concerns. Anonymity, therefore, serves as an important mechanism for promoting candid feedback and mitigating concerns about potential repercussions (19). Incorporating wellbeing education within the institutional wellbeing initiatives also reflects a thoughtful adaptation of global standards. Incorporating wellbeing education into institutional wellbeing initiatives also reflects a thoughtful adaptation of global standards, as studies found significant reductions in self-stigma after the intervention was delivered (20). Qatar’s focus on health and community wellbeing aligns with the ACGME-I requirement for promoting trainee wellness. Collaborations with the HR department’s wellbeing and staff engagement team can lead to programs and activities that are culturally relevant and accessible to all trainees. Furthermore, the provision of counseling services through an anonymous hotline represents a proactive approach to mental health, allowing trainees to seek help without the stigma that may exist in certain contexts. This system is especially vital in a region where discussing mental health issues might be culturally sensitive. Implementing an immediate triaging system ensures that trainees receive prompt support, adhering to ACGME-I’s standards for timely interventions. Overall, the glocalization of ACGME-I standards in Qatar training programs demonstrates a commitment to creating a nurturing educational environment that respects both international best practices and local cultural nuances.

#### Graduate medical education committee

At HMC, the ACGME-I requirement for institutional commitment to GME through governance, leadership, and resources was operationalized through the establishment of the Graduate Medical Education Committee (GMEC) as an integral component of institutional governance, with formal reporting lines to the Chief Medical Officer. This governance configuration reflects a glocalized interpretation of ACGME-I expectations, whereby international standards for independence and oversight were embedded within existing the institution decision-making structures. The GMEC members was composed of the Chief Medical Officer, leaders from Human Resources, Corporate Quality, and Patient Safety departments, as well as from medical universities in Qatar. This diverse membership ensured a holistic approach to decision-making and fostered strong connections among all stakeholders involved in GME.

#### Program personnel and resources

ACGME-I requires GME programs to be led by qualified program directors and supported by suitably trained faculty, with structured and ongoing faculty development. In HMC, program directors are appointed based on clearly defined qualifications aligned with international benchmarks, while allowing for contextual interpretation of professional credentials. Although American Board certification is commonly recognized within U. S.-based accreditation frameworks, it was not mandated in the Qatari context. Instead, locally and regionally recognized certifications—including the Arab Board and the Qatari Board—were accepted as equivalent, without compromising the rigor or credibility of appointments. This adaptation exemplifies glocalization by maintaining the *intent* of the ACGME-I requirement—ensuring capable and competent educational leadership—while contextualizing the *form* of credentialing to reflect regional professional standards and workforce realities. Nomination of program directors and core faculty follows a structured local governance process, whereby candidates are proposed by departmental leadership, reviewed by the DIO, and approved by the GMEC. Rather than mirroring U. S. full-time equivalent norms, protected time and delegated authority are determined according to institutional workload models and service demands, reflecting a locally responsive approach to resource allocation.

Faculty development initiatives aimed at combining international collaboration together with local capacity building, resulting in trained local faculty to deliver programs and foster peer education. These initiatives are systematically tailored to institutional and program needs, identified through ACGME-I surveys, compliance with ACGME-I data, annual notifications, and structured feedback from directors, faculty, and trainees. Several examples of faculty development programs are available, covering areas such as teaching, assessment, mentorship, and addressing biases, bullying, and harassment in medical education, all tailored to the local context. For example, faculty development sessions on bias, bullying, and harassment were contextualized through the use of locally relevant case studies while maintaining universal principles. Recognizing that perceptions of bullying vary across cultural contexts, the inclusion of local examples improved the relevance and authenticity of the educational content. These data-driven processes ensure alignment with global benchmarks while addressing local educational priorities, resulting in a sustainable faculty development system that integrates international expertise with contextual relevance.

#### Educational program

Program structure, curriculum design, and assessment methods remained internationally credible while being responsive to national needs. Several residency and fellowship programs in Qatar were adapted beyond the standard length commonly observed in U. S.-based programs. In multiple specialties, training duration was extended by an additional year to align with local and regional board certification requirements as well as advancement and promotion criteria within the training programs. For example, internal medicine and family medicine residency programs typically last 3 years, as per ACGME-I standards, while candidates in Qatar sit for the Arab Board, which mandates a four-year training period. Consequently, the duration of residency programs was extended to 4 years. This adaptation reflects a glocalized interpretation of ACGME-I standards, whereby the emphasis on competency attainment and readiness for independent practice was preserved, while program length was modified to meet local certification pathways and workforce expectations.

All residency and fellowship curricula were designed to align with the six ACGME core competencies, using a locally developed template to ensure all requirements are met. The establishment of new residency or fellowship programs required formal justification based on institutional service needs, supported by data on patient volumes, case mix, and projected workforce demands. This needs assessment was communicated through departmental leadership, with the chair providing both strategic justification and the necessary support at both departmental and institutional levels prior to curriculum approval. The educational curriculum, including content and teaching strategies, was designed to reflect local patient demographics, disease patterns, and cultural norms, with a focus on communication skills and professionalism, emphasizing ethical sensitivity, cultural respect, and effective interactions with a diverse, multilingual patient population. These principles were put into practice through locally developed educational activities, such as culturally relevant case scenarios, simulation exercises that represented common patient interactions in Qatar, and multilingual communication workshops. By doing so, global standards for professionalism and interpersonal skills were embodied in education that was meaningful for the local context.

In addition, clinical rotation assignments were adjusted in response to service and learner needs. For example, within the Emergency Medicine and Internal Medicine residency programs, more senior residents were strategically allocated to clinical areas requiring advanced communication and professionalism skills. This approach aimed to enhance patient satisfaction while ensuring appropriate support and safeguarding junior trainees. Additional research and specialty-specific rotations were introduced in selected programs to address departmental and workforce needs. In regard to procedural training and assessment, adjustments were made to the case and procedure log requirements. After receiving approval from ACGME-I, the Review Committees-International sanctioned variances in specific procedural requirements to align with the realities of local patient populations and service patterns. For instance, the required number of gynecological and urological procedures was reduced because it was found that certain case types were not sufficiently common within the Qatari context for trainees to meet the thresholds used in the U. S. This modification maintained the competency-based focus of procedural assessment while ensuring that the training was feasible and fair within the local environment.

#### Evaluation

The ACGME-I mandates thorough formative and summative evaluations for residents and fellows, overseen by Clinical Competency Committees (CCCs). In HMC, these requirements were adapted through a glocalization approach, blending competency-based assessment with local clinical practices and educational contexts. Program Directors establish CCCs by appointing qualified faculty members and defining their roles, structure, and responsibilities in alignment with ACGME-I requirements, ensuring representation of clinicians who have direct experience evaluating residents. In order to strengthen the effectiveness of CCC meetings, Program Directors develop structured evaluation checklists aligned with Milestones at the same time modified based on feedback from faculty and trainees, conducting pre-meeting calibration exercises to promote inter-rater reliability, and providing ongoing faculty development to ensure consistent and informed competency-based judgments. This involved adapting these evaluation checklists to local patient demographics and departmental practices (e.g., in-training and board examinations), ensuring competency assessments reflect real clinical contexts rather than abstract standards. This approach exemplifies glocalization by preserving standardized frameworks while enabling local relevance and flexibility.

Furthermore, evaluation tools were selectively adapted across specialties to reflect differences in clinical exposure and service delivery models. Programs such as Hematology fellowship and Pathology residency programs—where training is characterized by significant laboratory-based learning and consultative practice—developed supplementary assessment tools tailored to their educational context. In addition, all milestone checklists were locally developed by the program leaders with the support of ACGME-I faculty, ensuring meaningful assessment of competency development in local settings without compromising accreditation standards. This ongoing feedback loop highlights glocalization as a dynamic process rather than a static adjustment.

The Program Evaluation Committee (PEC) supports continuous program improvement. In accordance with ACGME-I requirements, program directors appoint PEC members including resident representatives that are responsible for planning, developing, implementing, and evaluating the overall educational program. In the context of HMC, PEC action plans are informed not only by accreditation metrics but also by local workforce needs, service pressures, and institutional priorities. Adaptations of curricula, assessment tools, rotations, and educational activities are revised based on these needs as discussed above. The findings from program evaluations are discussed within established governance structures, including GMEC meetings. This process reinforces accountability while allowing programs the flexibility to address identified gaps or opportunities.

## Conclusion

ACGME International accreditation represents a powerful opportunity to elevate graduate medical education worldwide. Accreditation can be both globally rigorous and locally relevant, ensuring sustainable excellence in health professions education. Using the Qatar experience as a case example, this paper demonstrates that institutional motivations for pursuing accreditation extended beyond compliance and quality assurance to include the development of a coordinated graduate medical education system, workforce capacity building, and alignment with national health-care priorities. Within HMC, accreditation was implemented in a stepwise glocalization framework in which international standards were adapted to local educational, cultural, and clinical realities through institution-wide governance, faculty development, context-responsive assessment systems, trainee support mechanisms, and continuous stakeholder engagement. This approach facilitated the consistent application of accreditation standards across a large and diverse training environment while remaining responsive to local workforce needs, cultural values, and clinical practice realities. Ultimately, Qatar’s experience illustrates that the impact of international accreditation depends not only on adherence to global standards but also on their thoughtful integration within local contexts. By doing so, accreditation becomes not merely a mechanism of compliance but a catalyst for innovation, equity, capacity building, and culturally attuned excellence in health professions education.
